# Cell Suspensions of *Cannabis sativa* (var. Futura): Effect of Elicitation on Metabolite Content and Antioxidant Activity

**DOI:** 10.3390/molecules24224056

**Published:** 2019-11-09

**Authors:** Damiano Gabotti, Franca Locatelli, Erica Cusano, Elena Baldoni, Annamaria Genga, Laura Pucci, Roberto Consonni, Monica Mattana

**Affiliations:** 1 Institute of Agricultural Biology and Biotechnology, National Research Council, Via Bassini 15, 20133 Milan, Italy; damiano.gabotti@gmail.com (D.G.); locatelli@ibba.cnr.it (F.L.); baldoni@ibba.cnr.it (E.B.); genga@ibba.cnr.it (A.G.); 2 Institute of Chemical Sciences and Technologies “Giulio Natta”, Lab. NMR, National Research Council, Via Bassini 15, 20133 Milan, Italy; cusano@cnr.it (E.C.); consonni@cnr.it (R.C.); 3 Institute of Agricultural Biology and Biotechnology, National Research Council, Via Moruzzi 1, 56124 Pisa, Italy; pucci@ibba.cnr.it

**Keywords:** *Cannabis sativa*, elicitation, plant cell cultures, metabolite profiling, NMR spectroscopy, secondary metabolism

## Abstract

*Cannabis sativa* L. is one of the most-studied species for its phytochemistry due to the abundance of secondary metabolites, including cannabinoids, terpenes and phenolic compounds. In the last decade, fiber-type hemp varieties have received interest for the production of many specialized secondary metabolites derived from the phenylpropanoid pathway. The interest in these molecules is due to their antioxidant activity. Since secondary metabolite synthesis occurs at a very low level in plants, the aim of this study was to develop a strategy to increase the production of such compounds and to elucidate the biochemical pathways involved. Therefore, cell suspensions of industrial hemp (*C. sativa* L. var. Futura) were produced, and an advantageous elicitation strategy (methyl jasmonate, MeJA) in combination with precursor feeding (tyrosine, Tyr) was developed. The activity and expression of phenylalanine ammonia-lyase (PAL) and tyrosine aminotransferase (TAT) increased upon treatment. Through ^1^H-NMR analyses, some aromatic compounds were identified, including, for the first time, 4-hydroxyphenylpyruvate (4-HPP) in addition to tyrosol. The 4-day MeJA+Tyr elicited samples showed a 51% increase in the in vitro assay (2,2-diphenyl-1-picrylhydrazyl, DPPH) radical scavenging activity relative to the control and a 80% increase in the cellular antioxidant activity estimated on an ex vivo model of human erythrocytes. Our results outline the active metabolic pathways and the antioxidant properties of hemp cell extracts under the effect of specific elicitors.

## 1. Introduction

*Cannabis sativa* L. is one of the oldest plants known in medicine and as a fiber crop and one of the most studied species for its phytochemistry [1]. Hemp is characterized by an extremely complex secondary metabolism with compounds belonging to different classes: terpenoids, alkaloids, stilbenoids, quinones and specific metabolites, the cannabinoids [2]. The hemp varieties can be distinguished into drug-type and fiber-type varieties based on their tetrahydrocannabinol (THC) content, with the THC content being high in the drug-type and low in the fiber type varieties [3]. Most studies have focused on drug-type *C. sativa*, which has been investigated for the presence of cannabinoids with pharmacological activity [4]. To date, approximately 120 cannabinoids have been isolated [5,6]. In recent decades, there has been an emerging interest in the fiber-type varieties approved for commercial use by the European Union. Indeed, these hemp plants produce many other specialized secondary metabolites that are directly or indirectly derived from the phenylpropanoid pathway [7,8]. The interest in these molecules is due to their bioactivities on human health, especially their antioxidant potential, and their ability to reduce some chronic diseases, such as cardiovascular and neurodegenerative problems [8]. Moreover, the search for natural antioxidants to be used for other purposes, such as bioactive packaging, is a field that has been attracting great interest from the scientific community because of the lower toxicity and higher safety compared to synthetic antioxidants [9]. The utilization of plant extracts incorporated into a film as active packaging has been investigated in several food applications, such as meat or fish [10].

Secondary metabolites are usually produced at very low levels, less than 1% dry weight [11]. Moreover, even among the plant material belonging to the same species, the chemical content may vary. This lack of reproducibility may depend on many factors, such as genetic variability, differences in growing conditions, age, tissue type and storage conditions [3]. For these reasons, research on phytochemicals is mainly directed to find alternative strategies for large-scale production. Chemical synthesis has become a feasible approach for obtaining plant natural compounds with simple molecular structures; however, it is often not economically convenient to obtain products with more complex structures or specific stereochemical requirements [12]. Moreover, the use of harsh solvents makes these chemical procedures environmentally unfavorable. The drawbacks of chemical synthesis could be overcome by the use of a biological system. The plant cell culture approach represents an attractive alternative to whole plants since it overcomes frequent agricultural problems such as seasonal dependency, length of the plant life cycle, and adverse environmental factors [13]. Furthermore, in cell culture systems, the production of secondary metabolites can be enhanced by treatment with elicitors and precursor feeding. An elicitor may be defined as a substance that, when introduced in small concentrations to a living cell system, initiates or improves the biosynthesis of specific compounds [14]. According to their nature, elicitors can be divided into abiotic and biotic. Abiotic elicitors can be considered substances of non-biological origin, which predominantly consist of inorganic compounds such as salts or physical factors (high pH, UV light, extremes of temperature, fungicides, antibiotics, heavy metals, high salt concentrations, etc.), whereas biotic elicitors are substances with a biological origin, including polysaccharides derived from plant cell walls (pectin or cellulose) or microorganisms (chitin or glucans). A further advantage to stimulate secondary metabolite production is represented by precursor feeding combined with elicitation, based on the idea that introduction of an intermediate compound of a biosynthetic route can increase the yield of the final product. Currently, many highly valuable metabolites belonging to polyphenols, alkaloids, terpenes, and lignans are commercially produced via plant cell cultures [15]. Nevertheless, some problems still have to be solved with this technology, resulting from the instability of the cell lines, low yields, and scale-up processes. As far as cannabinoids are concerned, callus and cell suspension cultures are not able to produce these kinds of molecules probably because their synthesis is restricted to specialized tissues, the trichomes [16]. However, other interesting secondary metabolites are synthesized by different hemp plant tissues and could be obtained by plant cell cultures [15,17,18]. In a previous study on several hemp varieties, we reported the presence of flavonoid compounds from leaves, mainly apigenin- and luteolin-7-O-glucoside [7]. The aim of this study was to evaluate the ability of hemp cell suspensions to synthesize valuable metabolites and to increase their production using different kinds of elicitors. The metabolite production was followed by NMR spectroscopy, and the metabolic pathways involved in the synthesis of the major identified compounds were also investigated following the activities of the enzymes phenylalanine ammonia-lyase (PAL) and tyrosine aminotransferase (TAT) and their expression profiles. Moreover, the antioxidant abilities of the cell suspension extracts were estimated through in vitro and ex vivo assays to verify whether the hemp suspension cell system showed antioxidant activity.

## 2. Results

### 2.1. Cell Suspension Growth and Elicitor Treatments

**Figure 1:**
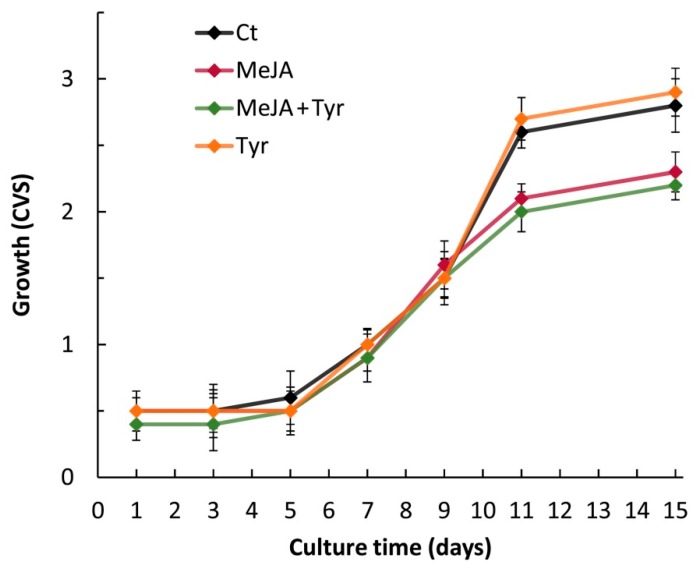
Growth curve of hemp cell suspensions (var. Futura) not elicited (Control, Ct), elicited with methyl jasmonate (MeJA), MeJA + tyrosine (MeJA + Tyr), or treated with tyrosine (Tyr). CVS: cell volume after sedimentation. Each value represents the average of five biological replicates ± SD.

*C. sativa* L. (var. Futura) cell suspensions were grown for a maximum of 15 days. During this period, the cells showed a typical growth curve, with the lag phase up to the 5th day and the exponential phase from the 5th to the 11th day (Figure 1). During the lag and exponential phases, the cell viability was greater than 88%; then, a decrease in the growth rate was observed together with the browning of the cultures and the development of clumps, both of which are symptoms of ageing (data not shown). The cell volume after sedimentation (CVS) was used to follow the growth in each flask. Upon inoculum in liquid medium, the cell biomass reached the maximum growth rate between days 5–11, as measured both by CVS (Figure 1, Ct) and by fresh weight (data not shown).

The elicitor treatments were applied to the hemp cells after cell growth entered the exponential growth phase (5th/7th day), as secondary metabolites are mostly synthesized during this period [19,20].

Before starting elicitor treatment, the cell viability, as estimated by Evans Blue dye, ranged between 80 and 90%. This percentage was considered mandatory to proceed with the treatments.

Several elicitor treatments were tested: salicylic acid (SA), chitosan (CHT), jasmonic acid (JA), methyl jasmonate (MeJA), and a combination of MeJA and precursor feeding with L-phenylalanine (Phe) or L-tyrosine (Tyr). Cell growth was differentially affected by the different treatments. Namely, CHT and SA did not impair either the growth rate or the cell viability (data not shown), whereas JA and MeJA caused a decrease in the growth rate. The MeJA-elicited cells showed a 20–23% decrease in the growth rate after 4 days of treatment (Figure 1). A similar decrease was observed with JA (data not shown). Conversely, the addition of Tyr alone did not have any inhibitory effect on the growth rate (Figure 1).

**Figure 2:**
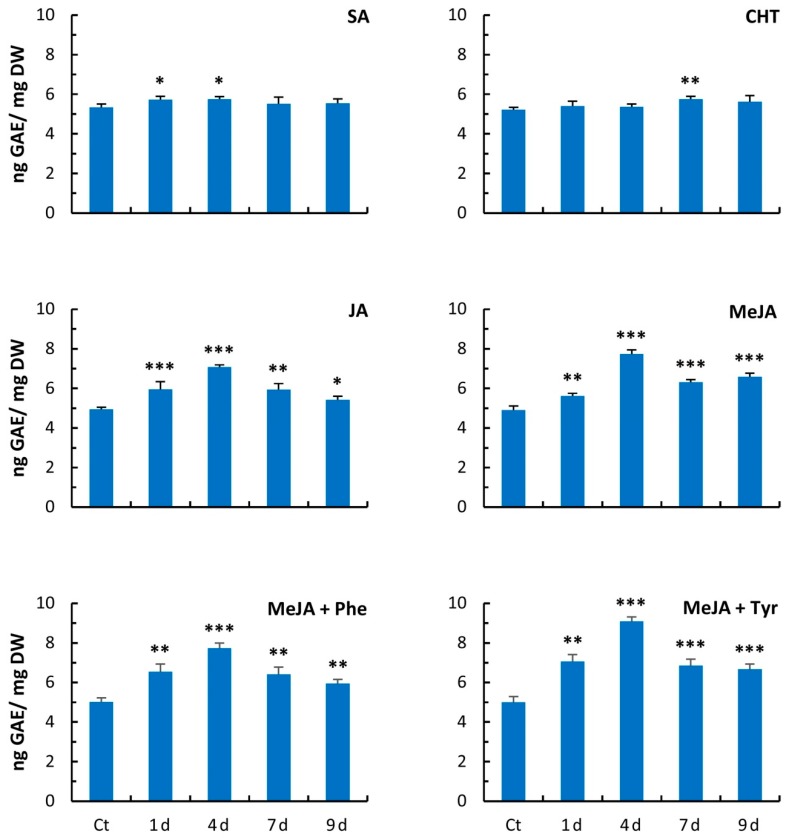
Total phenolic accumulation in hemp suspension cultures during nine days of elicitation. Each bar represents the average of three biological replicates ± SD. SA: salicylic acid; CHT: chitosan; JA: jasmonic acid; MeJA: methyl jasmonic acid; MeJA + Phe: MeJA + phenylalanine; MeJA + Tyr: MeJA + tyrosine. Ct: control; d: days of elicitation; DW: dry weight; GAE: gallic acid equivalent. Comparisons of differences between the means of the treated and Ct samples were performed using Student’s *t*-tests (* *p* ≤ 0.05; ** *p* ≤ 0.01; *** *p* ≤ 0.001).

To evaluate which elicitor was more effective at enhancing secondary metabolite production in hemp cell cultures, the total phenolic acid accumulation was measured during a time course experiment that lasted nine days after elicitation. As reported in Figure 2, SA and CHT treatments gave rise to only a slight increase in total phenolic accumulation, whereas JA and MeJA showed a more significant effect. In particular, the strongest effect of the two elicitors was evident on day 4 after addition, accounting for approximately 42% and 52% more phenolic accumulation, respectively (Figure 2). Based on these results, a 4-day elicitation with MeJA was chosen for subsequent experiments, where a combination of MeJA and precursor feeding using either Phe or Tyr was adopted. Namely, the combination of MeJA with Phe during 4 days of treatment led to a 56% increase in total phenolics compared to the control, whereas the combination of MeJA with Tyr amounted to 82% more phenolics than the control, thus leading to a further increase of 20% compared to the treatment with only MeJA. Based on these data, the 4-day treatment with a combination of MeJA + Tyr was chosen for all subsequent experiments.

### 2.2. NMR Metabolite Profiling

**Figure 3:**
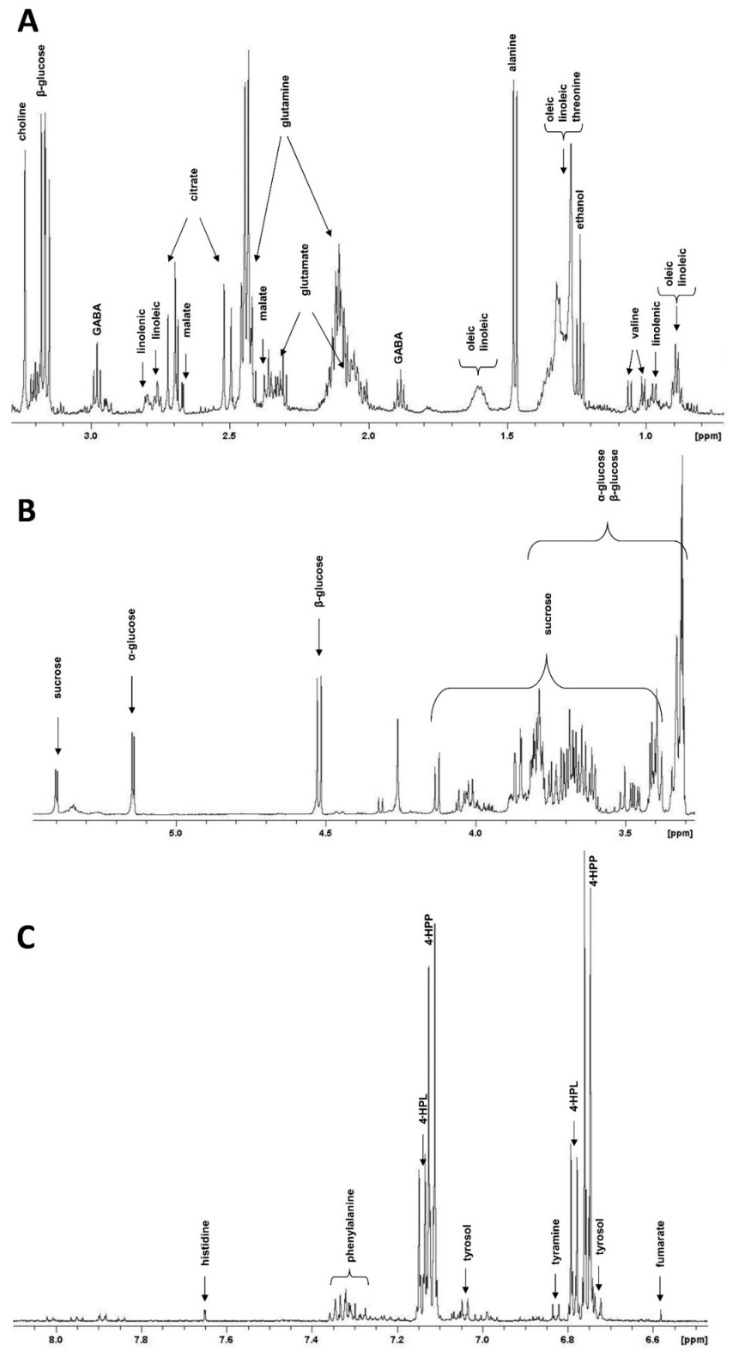
Aliphatic (**A**), anomeric (**B**), and aromatic (**C**) regions of ^1^H-NMR spectra of the methanol/water extracts from hemp cell suspension culture control samples. The resonance assignments of the main compounds are reported. GABA: γ-aminobutyric acid; 4-HPP: 4-hydroxyphenylpyruvate; 4-HPL: 4-hydroxyphenyllactate.

NMR metabolite profiling of hemp cell suspension cultures was used to identify secondary metabolic pathways that are induced during elicitation and that may be linked with the above-described total phenolic acid accumulation. To this end, the methanol/water extracts from the control and 4-day MeJA + Tyr treated cells were investigated by NMR spectroscopy (Figure 3).

The aliphatic region of the ^1^H-NMR spectra of the extracts from the control samples showed the presence of amino acids (alanine, γ-aminobutyric acid (GABA), glutamate, glutamine, threonine, valine), fatty acids (oleic, linoleic, and linolenic), and other organic compounds such as citrate, malate, and choline (Figure 3A). In the anomeric region, signals related to α-glucose, β-glucose, and sucrose were detected (Figure 3B).

In the aromatic region, the presence of fumarate, histidine, phenylalanine, 4-hydroxyphenylpyruvate (4-HPP), 4-hydroxyphenyllactate (4-HPL), 2-(4-hydroxyphenyl)ethanol (tyrosol), and 2-(4-hydroxyphenyl)ethanolamine (tyramine) are highlighted (Figure 3C). In particular, ^1^H-NMR signals due to 4-HPP and 4-HPL were prevalent with respect to the other aromatic compounds.

The main differences between the elicited cells and control samples were observed in the aromatic region. In particular, in the elicited samples, the signal intensities of 4-HPP and 4-HPL, after normalization performed with the methanol integral, showed a 2.84- and a 2.65-fold increase compared to the control, respectively (Table 1). The elicitation treatment also produced a 3.3-fold increase in tyrosol and a 1.77-fold increase in tyramine content.

**Figure 4:**
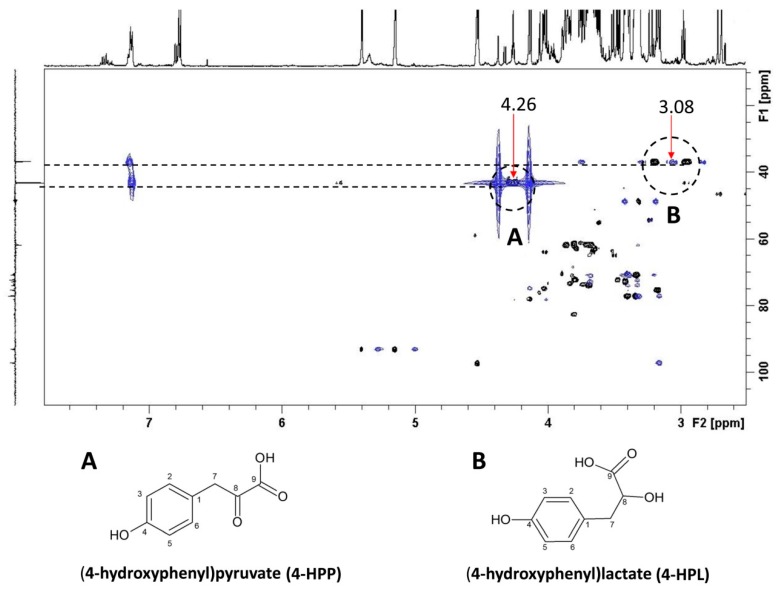
Spectrum overlay of the Heteronuclear Multiple Bond Correlation (HMBC, blue) and Heteronuclear Single Quantum Coherence (HSQC, black) spectra and Distortionless Enhancement by Polarization Transfer (^13^C DEPT) spectra included as F1 projection. Red arrows indicate the signals at 4.26 ppm and 3.08 ppm in the ^1^H frequency domain due to the ^13^C-enriched benzylic methylene of 4-HPP and 4-HPL, respectively.

Among the aromatic compounds, the resonance assignment was easily confirmed for phenylalanine (signals centered at 7.32 ppm), histidine (signal at 7.66 ppm), and fumarate (signal at 6.59 ppm) on the basis of the Biological Magnetic Resonance Data Bank database (BMRB, http://www.bmrb.wisc.edu/). Tyrosol (7.05 and 6.73 ppm) and tyramine (7.30 and 6.83 ppm) resonances were also confirmed by comparison with bidimensional heteronuclear experiments of standard compounds. The resonance assignment strategy of 4-HPP and 4-HPL was achieved by using a combination of two-dimensional (2D) homo- and heteronuclear NMR experiments performed on samples treated with MeJA and isotopically enriched Tyr (L-^13^C_3_-Tyrosine). The overlay of Heteronuclear Multiple Bond Correlation (HMBC, blue) and Heteronuclear Single Quantum Coherence (HSQC, black) spectra and Distortionless Enhancement by Polarization Transfer (DEPT) spectra included as an F1 projection (Figure 4) showed a strong direct correlation centered at 43.4 ppm and 4.26 ppm for the carbon and proton frequencies, respectively, due to the methylene signal, with two occasionally isochronous protons, as evidenced by the DEPT experiment. The heteronuclear long-range correlation in the HMBC experiment centered at 43.4 ppm and 4.26 ppm (indicated with a red arrow) with the 2′,6′ aromatic protons occurring at 7.12 ppm, confirmed the 4-HPP spin system assignment. The 4-HPL spin system assignment was performed by the same strategy. Figure 4 reports the direct ^13^C-^1^H correlation at 37 ppm and 2.97 ppm and 3.21 ppm (indicated with a red arrow centered at 3.08 ppm) for the methylene group of 4-HPL with two diastereotopic protons. The long-range heteronuclear correlation, centered at 37 ppm with the 2′,6′ aromatic protons at 7.15 ppm and the aliphatic protons at 3.75 ppm, confirmed the spin system assignment. Additionally, the monodimensional DEPT experiment confirmed signals at 43.4 and 37 ppm as methylene carbons, being in the opposite phase with respect to the other methyne signals in the aromatic region; the strong intensity of the two direct correlations observed in the HSQC spectrum confirmed that 4-HPP and 4-HPL were derived from the metabolism of ^13^C-enriched tyrosine.

### 2.3. Free Radical Scavenging Activity

**Figure 5:**
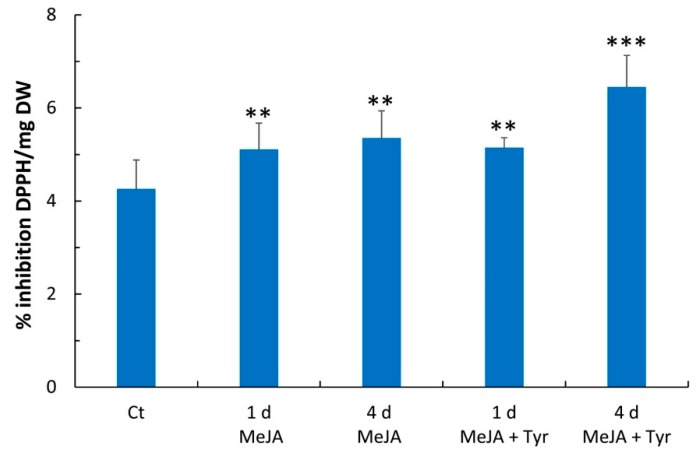
DPPH radical scavenging activity of the extracts from hemp cell suspensions subjected to different elicitation treatments. Ct: control; 1 d: 1 day; 4 d: 4 days; MeJA: methyl jasmonate; Tyr: tyrosine; DPPH: 2,2-diphenyl-1-picrylhydrazyl; DW: dry weight. Comparisons of differences between the means of the treated and Ct samples were performed using Student’s *t*-tests (* *p* ≤ 0.05; ** *p* ≤ 0.01; *** *p* ≤ 0.001). Each value represents the average of three biological replicates ± SD.

The 2,2-diphenyl-1-picrylhydrazyl (DPPH) method was used to estimate the antioxidant activity of the cell extracts before and after elicitation with MeJA and MeJA + Tyr. The treatment with MeJA led to an increase in scavenging activity of 20% and 26% after 1 day and 4 days of elicitation, respectively (Figure 5). The treatment with MeJA + Tyr produced a stronger effect on in vitro scavenging activity after 4 days of elicitation, resulting in a 51% increase relative to the control (Figure 5).

The antioxidant capacity results also correlated with the corresponding phenolic compound concentrations determined by the Folin-Ciocalteu method, giving rise to a correlation coefficient of R = 0.9299.

The biological effects of the cell extracts were also evaluated on an ex vivo model of human erythrocytes under oxidative insult in terms of both cellular antioxidant activity (CAA) and oxidative hemolysis inhibition.

**Figure 6:**
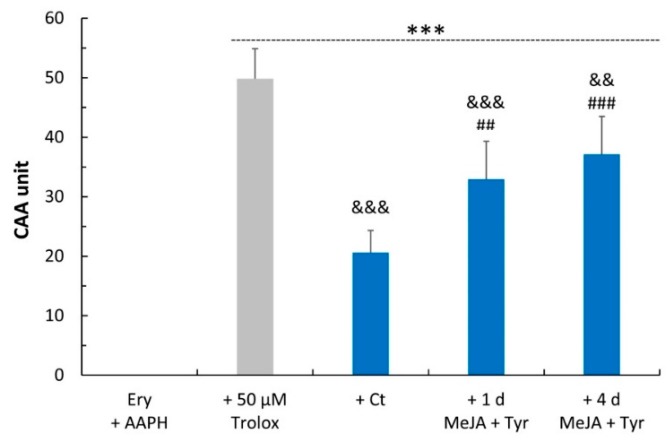
Cellular antioxidant activity (CAA) of hemp cell extracts evaluated in an ex vivo human erythrocyte system. Trolox was used as a reference standard. Values represent the mean ± SD of five measurements. Ery + AAPH: erythrocytes exposed to AAPH; Ct: control; 1 d: 1 day; 4 d: 4 days; MeJA: methyl jasmonate; Tyr: tyrosine. One-way ANOVA with Bonferroni’s post hoc test was applied: * indicates significance versus Ery + AAPH (*** *p* ≤ 0.001); ^#^ indicates significance versus the Ct sample (^##^ *p* ≤ 0.01; ^###^ *p* ≤ 0.001); and & indicates significance versus 50 μM Trolox (^&&^ *p* ≤ 0.01; ^&&&^ *p* ≤ 0.001).

The CAA was determined in human erythrocytes that were pre-treated for 1 h with 12.5 μg of cell extracts per mL of reaction volume and then exposed to a peroxyl radical generator (2,2′-azobis(2-amidinopropane) hydrochloride, AAPH), which causes oxidative stress. The effects of the cell extracts were compared to 50 μM Trolox, an analogue of vitamin E, which was used as a standard. As shown in Figure 6, the cell extracts after elicitation conferred stronger protection than non-treated samples, showing an antioxidant activity of 32.9 ± 6.4 CAA units after 1 day of elicitation and 37.1 ± 6.3 after 4 days of treatment, representing a 60% and 80% increase compared to the untreated cell samples.

**Figure 7:**
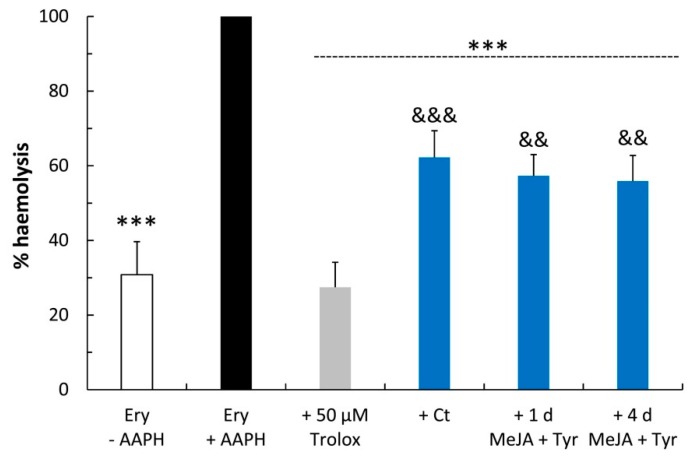
Anti-hemolytic activity of hemp cell extracts (12.5 μg/mL). Trolox was used as a reference standard. Values represent the mean ± SD of three measurements. Ery-AAPH: negative control without AAPH; Ery + AAPH: positive control with AAPH; Ct: control cell extracts; 1 d: 1 day; 4 d: 4 days; MeJA: methyl jasmonate; Tyr: tyrosine. One-way ANOVA with Bonferroni’s post hoc test was applied: * indicates significance versus Ery + AAPH (*** *p* ≤ 0.001); ^#^ indicates significance versus the Ct sample (^##^ *p* ≤ 0.01; ^###^ *p* ≤ 0.001); and & indicates significance versus 50 μM Trolox (^&&^ *p* ≤ 0.01; ^&&&^ *p* ≤ 0.001).

To conveniently evaluate the free radical-induced membrane damage and the antioxidant activity of the samples, hemp cell extracts were assayed in an AAPH-induced hemolysis model of human erythrocytes. This assay showed that all hemp extracts exerted significant erythrocyte hemolysis inhibition compared to the AAPH-treated erythrocytes (CNT), although they were less effective than 50 μM Trolox, as expected (Figure 7). In particular, the 1-day and 4-day elicited samples exhibited a higher anti-hemolytic effect compared to the untreated samples, which is in agreement with the CAA results in erythrocytes.

### 2.4. Effects of Elicitors on PAL and TAT Enzymatic Activities and Transcript Levels

PAL activity was investigated since PAL represents the crucial branch point between primary and secondary metabolism, being the first enzyme involved in phenylpropanoid derivative biosynthesis. In particular, in this study, the effect of elicitation and precursor feeding was considered. The PAL specific activity of non-elicited cells remained stable throughout the period of the experiment. The addition of MeJA for 1 and 4 days resulted in a 1.81- and 2.23-fold increase, respectively (Table 2). Elicitation with MeJA + Phe resulted in a 1.89-fold increase after 1 day and a 2.4-fold increase after 4 days of treatment compared to the control, whereas the addition of MeJA in combination with Tyr resulted in a 2.74-fold increase after 1 day and a 3.64-fold increase after 4 days relative to the control.

Since the NMR data highlighted the accumulation of 4-HPP, the activity of the TAT enzyme, which catalyzes Tyr deamination to generate 4-HPP, was also evaluated after elicitation with MeJA in combination with Phe or Tyr. As shown in Table 2, after 1 day of treatment with MeJA, the TAT activity was enhanced 1.48 times and the TAT activity was further enhanced 3.12 times after 4 days. When MeJA was added along with Phe, the enzymatic activity increased 2.24-fold after 1 day and 4.47-fold after 4 days of treatment. A further increase in TAT activity equal to 3.72- and 8.28-fold was observed using MeJA and Tyr as precursor for 1 and 4 days, respectively.

**Figure 8:**
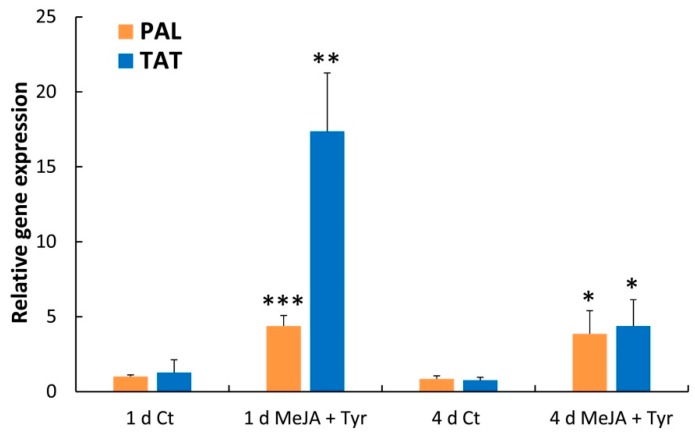
Expression analysis of the *PAL* and *TAT* genes from hemp cell suspensions after 1 and 4 days of treatment with MeJA + Tyr. Values represent the mean ± SD of three biological replicates. Comparisons of differences between the means of the treated and Ct samples were performed using Student’s *t*-tests (* *p* ≤ 0.05; ** *p* ≤ 0.01; *** *p* ≤ 0.001).

Considering that the PAL and TAT enzymes showed the highest increase after MeJA + Tyr elicitation, a quantitative expression analysis (qPCR) of *PAL* and *TAT* genes was performed on these samples. As shown in Figure 8, the expression of both genes was induced by this treatment. In particular, *PAL* gene expression was enhanced by approximately four-fold compared to the control samples after both 1 and 4 days of elicitation. The *TAT* gene expression was strongly induced (approximately 11-fold) after 1 day of elicitation compared to the control samples, whereas a lower induction was observed after 4 days of elicitation (approximately five-fold).

## 3. Discussion

Elicitation has been widely used to enhance secondary metabolism in plant cell suspension cultures [21]. In this work, several compounds were tested on cell suspensions obtained from the industrial hemp variety Futura to identify the best elicitor treatment that is able to induce secondary metabolism and to elucidate the metabolic pathways activated by the elicitation. The elicitors were chosen based on results achieved both on cannabis cell cultures [18] and on other species ([19] and references therein). Namely, CHT, SA, JA, and MeJA were tested in this study, as well as a combination of elicitation and precursor feeding. The elicitor treatments were performed during the exponential growth phase of cell suspensions between the 5th and 11th day. Indeed, it has been reported that secondary metabolites are mostly synthesized after the cell growth enters into the exponential phase, probably because during the lag phase, the plant cells need to adjust to the new environment [20,22]. Among the elicitors used, MeJA was the most effective, enhancing the polyphenol content by 52% over the control cells after 4 days of treatment. However, the biomass of cell cultures showed a reduction in growth rate upon MeJA supplementation. Several authors have highlighted that JA and MeJA treatments, although they lead to an increase in secondary metabolite accumulation, cause stunted growth in both plants and cell cultures through the suppression of mitosis [23,24,25]. It has also been reported that in plant cell cultures, precursor feeding in combination with elicitor treatments can enhance secondary metabolite synthesis. Therefore, the hemp cell suspensions were treated with MeJA + Phe or with MeJA + Tyr. Phe was chosen because it represents the substrate of PAL, the first committed enzyme of secondary metabolism, and Tyr was chosen since it is the precursor for thousands of specialized compounds [26,27]. Based on the amount of phenols that accumulated, the best combination proved to be MeJA + Tyr during 4 days of treatment, with an 82% phenol increase over the control cells. Likewise, MeJA + Phe produced an accumulation of phenols higher than the untreated samples (+ 56%) but lower than the MeJA + Tyr treatment. These data are in agreement with those reported for cell suspensions of other species such as *Withania somnifera*, *Papaver bracteatum, Catharanthus roseus*, and *Vitis vinifera* [26,28,29].

From the analyses of the aromatic region of the NMR spectra, the main compounds detected were 4-HPP and 4-HPL (here described for the first time in hemp cell suspension system), in addition to tyrosol and tyramine. Moreover, the levels of all these compounds were increased by the elicitation treatment with MeJA + Tyr. These results suggested that two biosynthetic pathways are mainly active in cultured hemp cells and that these pathways are enhanced by the elicitor/precursor treatment. The first is a Tyr-derived pathway leading to 4-HPP and 4-HPL, the two major compounds highlighted by the NMR spectra; the second is the pathway leading to tyrosol. As far as the first pathway is concerned, the elicitation treatment enhanced the amount of 4-HPP and 4-HPL by approximately three-fold with respect to the untreated samples. Tyrosine deamination to 4-HPP, a reaction catalyzed by the TAT enzyme, represents the entry point of many Tyr-derived compounds, such as plastoquinone, tocopherols, rosmarinic acid, and benzylisoquinoline alkaloids [27,30]. Indeed, TAT activity has been detected in hemp cell cultures together with its gene expression, and both its activity and expression are induced by all the elicitation treatments performed, with MeJA + Tyr being the most effective. Even if Phe is not the direct substrate of TAT, induction of TAT activity was also observed in MeJA + Phe-treated cells. This induction was higher than that observed after treatment with MeJA alone. This effect might be attributed to the direct conversion of Phe to Tyr due to an aromatic amino acid aminotransferase, similar to that observed in *Atropa belladonna* [31,32]. These authors reported that the Tyr and Phe pathways are biochemically coupled by specific aminotransferases.

Moreover, the presence of 4-HPL, derived from the reduction of 4-HPP, indicates that the hemp cell suspensions could follow the route of rosmarinic acid biosynthesis [33]. Nevertheless, no rosmarinic acid was found, suggesting that the last enzymatic steps needed for its synthesis are lacking in the hemp cell system. Indeed, it has been reported that rosmarinic acid is the prominent secondary metabolite of the Lamiaceae and Boraginaceae plant families [34].

The second pathway that is active in hemp cell cultures is the one that leads to the synthesis of tyrosol. In fact, the concentration of tyrosol after 4 days of elicitation with MeJA + Tyr was three times higher than that of the control. Two different biosynthetic routes for the synthesis of tyrosol have been proposed: one involves the reaction catalyzed by PAL with Phe as the substrate, and the other involves the decarboxylation of Tyr to tyramine [35]. In our cell suspensions, both PAL gene expression and enzymatic activity were induced by all the elicitation treatments. In particular, treatment with MeJA + Phe was more effective than treatment with MeJA alone, and a further increase was achieved with MeJA + Tyr. This induction might be due in part to the intrinsic tyrosine ammonia lyase activity of the PAL enzyme and partly to a cytosolic aminotransferase able to link Tyr catabolism to Phe biosynthesis, which might be active in our cell system, as described in *Petunia hybrida* [36]. Regarding the second route towards tyrosol, a slight increase of tyramine, the product of the first reaction, was observed after elicitation with MeJA + Tyr.

The presence of tyrosol, an antioxidant compound mainly found in olive oil and in plants of the Rhodiola species, was described for the first time in cell suspensions of a drug-type hemp variety by Peč et al. [18]. These authors obtained two types of cell lines, green and greenish-brown cells, that produced more tyrosol than olive oil after JA elicitation. In our experiments, the 4-day elicited hemp cell suspensions accumulated 2.19 µg tyrosol/mg dry weight (DW), a concentration similar to that observed by Peč et al. [18]. These results confirm that the hemp cell cultures represent a good system for tyrosol production. Further optimization could be achieved through suitable selection of genotypes and elicitation strategies.

The presence of the four aromatic compounds might be responsible for the antioxidant activity observed in hemp cell extracts. Indeed, our results on the hemp cell extracts showed an in vitro free radical scavenging activity increase with the use of specific elicitors and with the time of elicitation as estimated by the DPPH assay. The correlation observed between the DPPH assay and total phenolic content indicates that these compounds significantly contribute to the antioxidant potential of the hemp cell extracts, which is in agreement with previous papers reporting the link between phenolic content and antioxidant potential [37,38].

Moreover, the elicited cell extracts exhibited antioxidant activity on an ex vivo model of human erythrocytes under oxidative stress that was approximately double that of the untreated samples.

Taken together, our results outline the metabolic pathways that are active in the cell suspension of the hemp variety Futura under specific elicitation treatment and highlight the antioxidant properties of these cell extracts. Phenolic compounds from plant sources have been receiving great attention as natural antioxidants for many applications, such as pharmaceuticals, dyes, nutraceuticals, fragrances, flavors and pesticides. Currently, they are receiving great attention as natural antioxidants for food preservation, since concerns are increasing over the safety of synthetic preservatives. An example is the production of active food packaging through the incorporation of antioxidant compounds into the packaging material. Recently, active packaging using chitosan and kombucha tea or olive leaf extracts has been demonstrated to protect fresh meat oxidation, extending the shelf life [39,40]. Whole hemp cell extracts could represent a good candidate for such a purpose.

## 4. Materials and Methods 

### 4.1. Plant Material and Cell Suspension Cultures

*C. sativa* L. (var. Futura) seeds were soaked in a 0.05% Tween 20 solution for 5 min, rinsed several times with sterile water, and incubated at 25 °C onto 3MM Whatman wet paper for 3-4 days until germination. Seedlings were sterilized for 30 sec in 70% ethanol followed by 5 min in 1:5 (*v*/*v*) commercial bleach. After several rinses in sterile water, seedlings were transferred on Murashige & Skoog (MS) agar medium [41], and after 2–3 weeks, leaves were used to induce cell cultures. For callus induction, leaf pieces were cultured on MS agar medium supplied with 0.5 μM 1-naphthaleneacetic acid (NAA), 1 μM thidiazuron (TDZ), 3% (*w*/*v*) sucrose, 0.8% agar (*w*/*v*), adjusted to pH 5.8 [42].

One month later, calli were transferred on fresh medium, subcultured every 3 weeks and maintained in the dark for several months. The cell suspension cultures were started by inoculating about 1 gr of friable callus in a 250 mL Erlenmeyer flask containing 50 mL of liquid medium and kept on a gyratory shaker at 110 rpm under a light intensity of 14–23.8 μmol/m^2^sec. To determine the cell growth, CVS was measured using 250 mL Erlenmeyer flasks with a graduated beak. This method was chosen as it has been shown to be a rapid and simple method for the routine estimation of cell biomass, without the destruction of cells [43]. Moreover, CVS is highly correlated with the fresh weight of cells [43]. 

### 4.2. Elicitor Treatments

Exponentially growing cells (5/7 days after subculturing) were treated with different elicitors: SA, CHT, JA, MeJA and a combination of MeJA + Phe or MeJA + Tyr. 

SA was dissolved in deionized water, filter sterilized, and added to the cell suspensions to the final concentration of 100 µM. CHT was dissolved in 0.1% acetic acid under continuous stirring; then, the pH was adjusted to 5.6 using 0.1 M NaOH. The stock solution (10 mg/mL) was kept at −20 °C and diluted, before use, to a final concentration of 100 µg/mL. JA and MeJA were dissolved in 100% ethanol, sterilized by filtration (0.22 µm) and added to the cultures to a final concentration of 100 µM. Cell suspensions supplemented with ethanol at the same final concentration were used as control. L-Tyr was added to the cell suspensions at a final concentration of 1 mM. The elicited and control cells were collected after 1, 4, 7, and 9 days after treatment, ground in a mortar under liquid nitrogen, freeze-dried, and stored at −80 °C until extraction.

### 4.3. Cell Viability

Cell viability was assessed by Evans blue (Sigma-Aldrich, St. Louis, MO, USA) vital exclusion dye as reported by Iriti et al. [44]. Cell suspensions were incubated for 10 min with 0.15 mg/mL of Evans blue in distilled water (1:1). The excess of stain was removed from the medium with distilled water, whereas the stain bound to dead cells was solubilized in 50% aqueous methanol containing 1% SDS and quantified spectrophotometrically by measuring the absorbance at 600 nm. Heat treated cells (10 min, 100 °C) were used as control of 100% cell death.

### 4.4. Metabolite Extraction and NMR Measurements

The freeze-dried samples were extracted in 80% methanol. Briefly, the dried fractions (10 mg) were dissolved in 1 mL of 80% methanol, vortexed, sonicated for 10 min, and extracted overnight on an orbital shaker at room temperature in the dark. Extracts were centrifuged for 20 min at 13,000× *g* and the clear supernatants were used for total phenolics determination and DPPH radical scavenging assay. The sample extraction procedure for NMR analysis was the same as above but methanol-d_4_ and KH_2_PO_4_ buffered in D_2_O at pH 6.0 were used [45]. The samples were centrifuged and 600 μL of the supernatant were directly analyzed. All NMR spectra (^1^H, ^13^C DEPT, Total Correlation Spectroscopy (TOCSY), HSQC, Heteronuclear two-Bond Correlation (H2BC), HMBC, and HSQC-TOCSY) were recorded on a Bruker DRX 600 spectrometer (Bruker Biospin GmbH Rheinstetten, Karlsruhe, Germany) operating at 14.1 T, equipped with a 5 mm probe and z axis gradient unit. Spectra were acquired at 300 K, with a spectral width of 10,000 Hz and 32 K data points. The residual water suppression was achieved by applying a presaturation scheme with low-power radiofrequency irradiation for 1.2 sec. A resolution enhancement function with an exponential multiplication of 0.5 Hz for the line broadening was applied. Spectra were referenced to the residual solvent signal at 3.31 and 49.0 ppm for proton and carbon dimension, respectively. All ^1^H NMR spectra were carefully phased and baseline-adjusted with the TOPSPIN 3.0 software (Bruker Biospin GmbH Rheinstetten, Karlsruhe, Germany). Spectra assignment was performed also with the aid of standard compounds (Sigma-Aldrich, St. Louis, MO, USA). L-Tyrosine-^13^C_3_ enriched was purchased by Sigma Aldrich (Sigma-Aldrich, St. Louis, MO, USA).

### 4.5. Assay of Total Phenolics

Total phenolic content of the samples was determined spectrophotometrically according to the Folin-Ciocalteu method [46], using gallic acid as a standard. The extract (20 μL) was mixed with 1.58 mL water and 0.1 mL Folin-Ciocalteu reagent previously diluted 1:10, incubated for 8 min, then 0.3 mL of 20% sodium carbonate solution were added and the reaction was incubated at room temperature for 2 h. The absorbance was measured at 765 nm against a reagent blank without extract. The results were expressed as ng of Gallic Acid Equivalent (GAE) per mg of dry weight. 

### 4.6. DPPH Radical Scavenging Assay

The effect of extracts on DPPH radical was determined following the method described by Cheng et al. [47]. A working solution of 0.208 mM fresh DPPH in methanol was made daily and mixed with different concentrations of the extracts. The reaction mixture was vortexed and left in the dark at room temperature for 30 min. The absorbance of the mixture was measured spectrophotometrically at 517 nm against a blank of 80% methanol; then, the ability to scavenge DPPH radical was calculated as follows:

(%) DPPH radical scavenging activity= [(A_ct_ − A_sa_)/A_ct_] × 100 (1)

where A_ct_ is the absorbance of DPPH radical + methanol and A_sa_ is that of DPPH radical + sample extract.

### 4.7. Cellular Antioxidant Activity (CAA) Assay in Red Blood Cells

Human erythrocytes were collected from healthy blood donors upon informed consent for the use of residual blood for research purposes, according to the regulations of “Fondazione G. Monasterio CNR-Regione Toscana”. Human blood samples from volunteers were collected in ethylenediaminetetraacetic acid (EDTA)-treated tubes and centrifuged for 10 min at 2300× *g* at 4 °C. Plasma and buffy coat were discarded and erythrocytes were washed twice with phosphate buffered saline (PBS), pH 7.4. The antioxidant activity sample extracts was evaluated in an ex vivo erythrocyte system as described by Frassinetti et al. [48]. The fluorescence was read at 485 nm excitation and 535 nm emission by using a VictorTM X3 Multilabel Plate Reader (Waltham, MA). Each value was expressed as follows:

CAA unit = 100 − (∫SA⁄∫CA) × 100(2)

where ∫SA is the integrated area of the sample curve and ∫CA is the integrated area of the control curve [49].

### 4.8. Erythrocyte Oxidative Hemolysis

The erythrocyte hemolysis was measured according to the method described by Mikstacka et al. [50] and the oxidative stress was generated by thermal decomposition of AAPH to peroxyl radicals. The erythrocyte oxidative hemolysis was spectrophotometrically evaluated at 540 nm as hemoglobin released in the supernatant. Control and blank samples were used and refer to erythrocytes exposed to AAPH or PBS, respectively. Each value was expressed as a percentage of hemolysis relative to the control.

### 4.9. PAL and TAT Enzymatic Assays

PAL: the freeze-dried samples were suspended in ice-cold extraction buffer containing 0.1 M phosphate buffer (pH 7.5), 0.1 mM EDTA, 1 mM DTT, 5 mM ascorbic acid, 1 mM PMSF and 0.15% *w*/*v* polyvinyl-pyrrolidone, vortexed and centrifuged at 13,000× *g* for 20 min at 4 °C and the supernatant was used for assaying PAL activity. PAL activity was determined spectrophotometrically at 290 nm measuring the appearance of cinnamic acid [7].

TAT: the freeze-dried samples were suspended in ice-cold extraction buffer containing 0.1 M phosphate buffer (pH 7.5), 0.1 mM EDTA, 1 mM DTT, 8 mM α-ketoglutarate, 0.2 mM pyridoxal-5-phosphate. After centrifugation at 6000× *g*, the supernatant was made 0.1% *v*/*v* with Triton X100, incubated 15 min on ice and then centrifuged at 13,000× *g* for 20 min at 4 °C. The supernatant was used to determine the enzyme activity following the method of [51]. The end product of the reaction was measured spectrophotometrically at 331 nm using the extinction coefficient of 24,900 L mol^−1^ cm^−1^.

The amount of soluble proteins was determined by the Bradford method using bovine serum albumin (BSA) as a standard [52]. Briefly, different amounts of the extracts were added to the Comassie dye reagent, incubated for 10 min at room temperature and the absorbance was measured at 595 nm. The sample concentration was determined by interpolation with a standard curve prepared with BSA.

### 4.10. RNA Extraction and Real Time PCR

Total RNA was extracted from 20 mg of lyophilized suspension cultures using the TRIzol^®^ RNA Purification Kit (Invitrogen, Carlsbad, CA, USA) following the manufacturer’s instructions. RNA purity was checked spectrophotometrically (NanoDrop 2000c, Thermo Fisher Scientific, Waltham, MA, USA), and only samples with a 260⁄280 nm ratio of absorbance comprised between 1.7 and 2.1 were further used. The integrity of the RNA was verified on agarose gels and stained with ethidium bromide. The cDNA was synthesized using 0.7 µg of total DNase I-treated RNA using the SuperScript III First-Strand Synthesis SuperMiX for quantitative RT-PCR (qRT-PCR), according to the manufacturer’s instructions (Invitrogen, Carlsbad, CA). Quantitative RT-PCR was performed using 20 μL triplicate reactions on a 7300 Real-Time PCR System (Applied Biosystems, USA) containing 5 μL of 1:10 diluted cDNA, a 300 nM final concentration of each primer, and 10 μL of SYBR Green PCR Master Mix (Applied Biosystems, USA). The cycling program was as follows: 50 °C for 2 min (1 cycle), 95 °C for 10 min (1 cycle), 95 °C for 30 sec, and 60 °C for 1 min (40 cycles). The primer sets were tested by dissociation curve analyses and verified for the absence of nonspecific amplification. The dissociation curves were constructed using the following conditions: denaturation at 95 °C for 15 sec, cooling to 60 °C for 30 sec, and then gradual heated at 0.01 °C/sec to a final temperature of 95 °C. For each primer pair, calibration curves were generated with different dilutions and were accepted when the correlation coefficient was ≥0.99 and the efficiency was 1 ± 0.1. Relative expression levels were calculated using the 2^−ΔΔCt^ method [53]. Negative controls without cDNA were routinely included. For the design of specific primers to produce amplicons of 150–250 bp, the Primer3 software (v. 0.4.0; http://primer3.wi.mit.edu) [54,55] was used.

A putative ubiquitin gene (AJ864397.1) of *C. sativa*, which was found to have a stable expression in all tested conditions, served as an endogenous reference. The following primers, producing an amplicon of 147 bp, were used: UBIfor 5′-GCCAGGATGGCAATGAAGTA-3′ and UBIrev 5′-GAGTCTGCTCAGCTCGAAGG-3′. Results were confirmed using actin as a second housekeeping gene (data not shown); for the amplification of the actin gene, the primers described by Stout et al. [56] were used. For the TAT gene, the following primers, producing an amplicon of 174 bp, were used: TATfor 5′-GGCCTGGTTTTCCCATTTAT-3′ and TATrev 5′-CATTCCCACAAGGATTACCG-3′. These primers were designed on a putative TAT sequence (FN13105.1), which was obtained from BLAT searches against the draft genome sequence of *C. sativa* var. Finola (http://genome.ccbr.utoronto.ca) using TAT sequences from Arabidopsis as a query. For the PAL gene, the primers for qRT-PCR described in [7] were used.

## Figures and Tables

**Table 1:** Quantification of aromatic compounds determined by ^1^H NMR analyses.

Compound	Ct (µg/mg DW)	4 d MeJA + Tyr (µg/mg DW)	Fold Increase
4-HPP	14.47	41.08	2.84
4-HPL	5.05	13.37	2.65
Tyrosol	0.66	2.19	3.32
Tyramine	1.02	1.80	1.77

Ct: control cell extracts; 4 d: 4 days; MeJA: methyl jasmonate; Tyr: tyrosine; 4-HPP: 4-hydroxyphenylpyruvate, 4-HPL: 4-hydroxyphenyllactate; DW: dry weight. Each value represents the average of three biological replicates.

**Table 2:** Specific activities of phenylalanine ammonia-lyase (PAL) and tyrosine aminotransferase (TAT) of hemp cell extracts before and after different elicitation treatments.

Sample	PAL Specific Activity (nmol mg^−1^ min^−1^)	TAT Specific Activity (nmol mg^−1^ min^−1^)
Ct	3.25 ± 0.80	0.58 ± 0.06
1 d MeJA	5.87 ± 0.71	0.86 ± 0.11
4 d MeJA	7.26 ± 0.33	1.81 ± 0.22
1 d MeJA + Phe	6.15 ± 0.20	1.30 ± 0.14
4 d MeJA + Phe	7.79 ± 0.39	2.59 ± 0.13
1 d MeJA + Tyr	8.90 ± 0.33	2.16 ± 0.24
4 d MeJA + Tyr	11.83 ± 0.75	4.80 ± 0.43

Values are expressed as the mean ± SD of three biological replicates. Ct: control cell extracts; 1 d: 1 day; 4 d: 4 days; MeJA: methyl jasmonate; Tyr: tyrosine.
